# Vaginal Cœliotomy

**Published:** 1899-10-28

**Authors:** 


### VAGINAL CŒLIOTOMY.

Among the more striking changes which have taken place in the practice of gynæcology is the steady extension of the employment of the vaginal route as a way of gaining access to the pelvic contents. Dr. Riddle Goffe,[1] Professor of Gynæcology to the New York Polyclinic Medical School, says that there are two vaginal incisions through which the cavity of the pelvis can be reached—one posterior to the cervix into Douglas' pouch, and the other anterior to the cervix, separating the bladder from the uterus, and opening up to view and to touch the entire contents of the pelvis. The latter, he says, is the incision which affords the greatest facility for operative procedures, and the one which he uses almost exclusively, although he sometimes makes a posterior incision in conjunction with it to afford additional opportunity for manipulation, or for purposes of drainage. The procedure consists in making a transverse incision through the anterior vaginal fornix and a longitudinal one at right angles to it through the entire length of the anterior vaginal wall. The bladder is then dissected from the uterus and also from the vaginal wall, to the extent of an inch or an inch and a half on each side of the longitudinal incision, and through the opening so made, whether in virgin or multipara, ample space is afforded for whatever radical or conservative work upon the uterus and its appendages may be indicated. When he began to use this incision he feared that it might lead to bladder complications, but he has never yet met with any symptoms which were referable to the urinary apparatus. As an exploratory operation it affords the means of acquiring definite information in regard to the entire contents of the pelvis; and, in regard to treatment, Dr. Goffe has used it, with three exceptions, for every case that has come before him for operation during the past three years, these cases embracing "every variety of disease, from simple retroversion with adhesions to prolapsed and cystic ovaries, unilateral and bilateral salpingitis, ectopic gestation, fibroid tumours of the uterus, and dermoid cysts." Among the advantages of this mode of access to the pelvis are that the healing process goes on unconsciously to the patient and without any more constitutional or local disturbance than normally attends a simple trachelorrhaphy; that the patient herself is not conscious of even having had an incision made; and that she does not bear upon her person any trace of a surgical operation. "Indeed, in many instances the wound heals so kindly that often an expert gynæcologist, even after the lapse of only a few months, might examine a patient and never suspect that an incision had been made."

[1] New York Med. News, Oct. 7.

